# Structural Evidence in *Plectroniella armata* (Rubiaceae) for Possible Material Exchange between Domatia and Mites

**DOI:** 10.1371/journal.pone.0039984

**Published:** 2012-07-05

**Authors:** Patricia M. Tilney, Abraham E. van Wyk, Chris F. van der Merwe

**Affiliations:** 1 Department of Botany and Plant Biotechnology, University of Johannesburg, Auckland Park, Johannesburg, South Africa; 2 Department of Plant Science, University of Pretoria, Pretoria, South Africa; 3 Laboratory for Microscopy and Microanalysis, University of Pretoria, Pretoria, South Africa; Roehampton University, United Kingdom

## Abstract

Domatia are small structures on the lower surface of a leaf, usually taking the form of cavities, pouches, domes with an opening, or hairs (or a combination of these), and located in the axils between the main veins. They are found in many dicotyledons including certain members of the Rubiaceae. As part of an ongoing study of selected southern African members of the tribe Vanguerieae of this family, their structure in transverse section was investigated. In some taxa, such as *Plectroniella armata*, light microscopic (LM) observations revealed large numbers of stomata in the domatia as well as a number of channel-like structures extending across the cuticle toward the cavity of the domatia. The cuticle of the epidermis lining the domatia also appeared thicker than in other parts of the leaves. The epidermis in *P. armata* was also examined using transmission electron microscopy (TEM). Domatia have been shown to house mainly mites, many of which are predatory or fungivorous, in a symbiotic (mutualistic) relationship with the plant. To date, much research has focussed on the role of domatia in providing shelter for various organisms, their eggs and their young. However, the TEM study revealed the apparent “channels” and thick cuticle seen under LM to be electron dense non-cellulosic branching fibrils within pronounced, often closely spaced cuticular folds. The functional significance of these fibrils and folds requires further investigation. Folding of cell walls and membranes at ultrastructural level is usually functionally associated with an increased surface area to facilitate active exchange of compounds/metabolites. This may indicate that translocation of substances and/or other forms of communication is possible between the domatium and its inhabitants. This therefore suggests a far more active role for the leaf in the symbiotic relationship than was previously thought. More work is required to test such a possibility.

## Introduction

Domatia are small structures found on the underside of the leaves of many woody plants, particularly those of humid tropical or subtropical regions [1]. They are most frequently present in the axils between the midrib and some of the secondary veins. These structures usually take the form of depressions/cavities of various types, pouches of leaf tissue, domes with an opening on the top, hair-tufts or, commonly, a combination of these [2]. Domatia are non-pathogenic and are frequently inhabited by mites [1].

The symbiotic relationship between domatia and mites (Acari) is generally recognised as being mutualistic. Since most mites associated with domatia are predatory (on phytophagous mites) or fungivorous, this could be beneficial to the plant by removing natural enemies that could cause damage to the plant directly or, indirectly, through the transmission of disease [3], [4]. Fungivorous mites may also help to clean leaf surfaces of epiphyllous fungal hyphae. In turn, domatia provide shelter for a variety of mites. Protection could be afforded to the mites from larger predators as well as from adverse environmental conditions. This would apply not only to the adults but also to their eggs and their young during development. Since some mites are known to require a high relative humidity to complete their life cycles, such shelters could play an important role in their survival. That domatia merely serve as physical shelters to mites has been the prevailing view on their possible function.

A generally neglected possibility when considering the functional role of domatia is that, in addition to physical protection, they also facilitate the exchange of compounds between plant and mite. As early as 1887, Lundström [5] proposed that mites may provide the plant with nutrients through their faeces. In their study on Australasian plants which included members of the Rubiaceae, O’Dowd and Willson [6] concluded that “the anatomy of domatia did not suggest any specialized role in nutrient breakdown or uptake or specialized features associated with insect traps”. In particular they found no structures or cells indicative of a glandular function, except in *Hebe townsendii*. However, there was uncertainty as to whether the structures in question were domatia or extrafloral nectaries. They also examined the activity of various enzymes through histochemical staining but did not demonstrate greater activity for any of the enzymes in the domatia than elsewhere. Glands have been reported in the domatia of several taxa [1]. In their studies, O’Dowd and Willson [6] found that the cuticles within the domatium and on the leaf surface were usually similar in thickness.

Domatia-bearing taxa are present in a wide range of dicotyledonous families and are particularly abundant in the Rubiaceae [2], [4]. In the tribe Vanguerieae of this family, they may or may not be present. They can be used for taxonomic purposes, e.g., [6], [7]. As part of an on-going study of selected southern African members of this tribe, their structure in transverse section using light microscopy (LM) was examined. In this communication, that of *Plectroniella armata*, a multi-stemmed shrub or small tree confined to subtropical/tropical savanna and thicket in the northeastern parts of South Africa, southern Mozambique and Swaziland, is reported. This contribution was motivated by the unusual appearance of the epidermal cells lining the domatia in this species. A transmission electron microscopy (TEM) study was also carried out to investigate it further. This revealed a highly folded cuticle with branching fibrils in the epidermis of the domatia suggestive of a pathway for the passage of substances between the plant and any mites in the domatia.

## Materials and Methods

Leaves of the tree *Plectroniella armata* (K.Schum.) Robyns [ = *Canthium armatum* (K.Schum.) Lantz] were collected in the Pretoria National Botanical Garden. The distribution of domatia and their association with mites were studied in fresh leaves under a stereomicroscope equipped with a fibre optics light source. To photograph the mites, they were first killed by placing a leaf briefly in a Petri dish containing a piece of cotton wool soaked with a few drops of chloroform.

For light microscopy, leaf material that had been preserved in FAA (formaldehyde: acetic acid: alcohol) at the University of Pretoria (*Van Wyk 3055* in PRU) was used. Small portions of leaves, consisting of domatia and surrounding tissue, as well as non-domatial parts from elsewhere on the blade, were removed and embedded in glycol methacrylate [8]. Transverse sections, 3–5 µm thick, were cut on an ultramicrotome and stained using the periodic acid Schiff/toluidine blue method [8]. Permanent mounts were made with Entellan (Product 7961, E. Merck, Darmstadt).

For the TEM study of the epidermis, FAA-preserved material or fresh leaves of *P. armata* fixed in glutaraldehyde, were used. Samples were all post-fixed with osmium tetraoxide and embedded in either LR White or epoxy resin. No contrasting with lead citrate and/or uranyl acetate was done. Monitor transverse sections, 0.5 µm thick and stained with 0.2% toluidine blue dissolved in 0.5% sodium carbonate, were made. Ultrathin transverse sections were cut and examined with a JEOL JEM 2100F transmission electron microscope.

## Results

**Figure 1:**
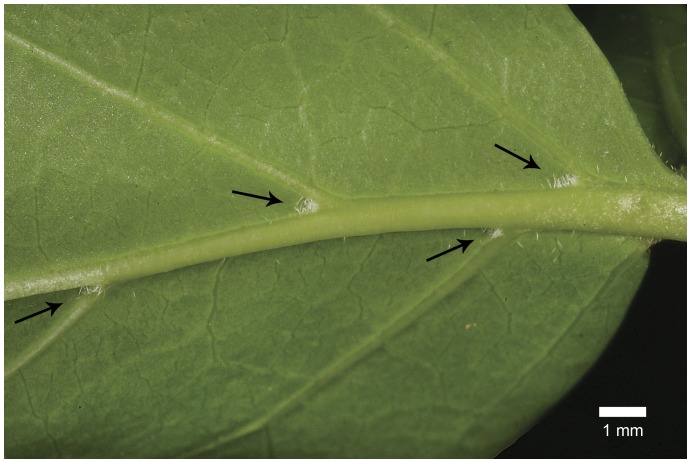
Leaf with domatia. Lower leaf surface of *Plectroniella armata* showing domatia (arrowed) in the axils of the midrib and primary lateral veins. Outwardly, the domatia appear as minute whitish hair-tufts, but the hairs are lining the entrance of a sac-like cavity that extends for up to about 0.5 mm into the leaf tissue.

**Figure 2:**
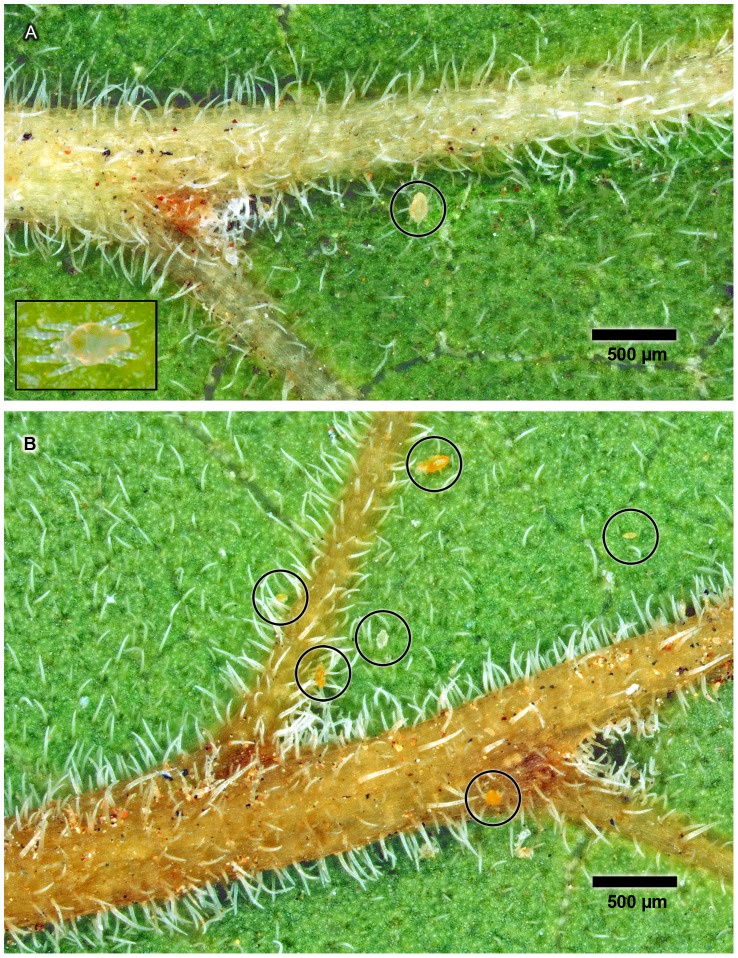
Domatia and associated mites. Stereomicrographs of the abaxial surface of fresh leaves to depict the relative sizes of the domatia and their associated tydeid mites (*Naudea richinda*). (A) Domatium in the axil of a primary lateral vein and a single mite (encircled). The mite is dead, having been exposed to chloroform fumes. When dead the eight legs are tucked underneath the body. Insert shows a live mite with body (idiosoma) approximately 180 µm long. Scale line applies to background image only. (B) Two domatia in the axils of primary lateral veins and several dead mites (encircled). The mites were either cream-coloured or pale orange; they varied in size and represent different developmental stages of the same species. Once the mites emerge from domatia they show rapid and incessant movement which makes them difficult to photograph, hence the need to kill them first.

Macroscopically the domatia of *P. armata* appear as minute whitish hair-tufts in the angles between the midrib and primary lateral veins on the abaxial leaf surfaces (Figure 1). Under the stereomicroscope several mites of the family Tydeidae (or superfamily Tydeoideae, family Iolinidae), *Naudea richinda* Meyer & Rodrigues (Ueckermann E., *pers. comm.*), were usually found on the leaf blade, mainly on the abaxial surface and especially in younger leaves. They were typically seen near the midrib and in the vicinity of domatia. The mites were minute, the bodies of the largest being about 180 µm long and 60 µm wide (Figure 2A, B). They moved actively over the leaf surface in a rather jerky manner, presumably in search of food (see Video S1). Because of the rapid and incessant movement it was very difficult to photograph live ones. Every now and then they would enter a domatium, structures they obviously use for shelter and rest.

**Figure 3:**
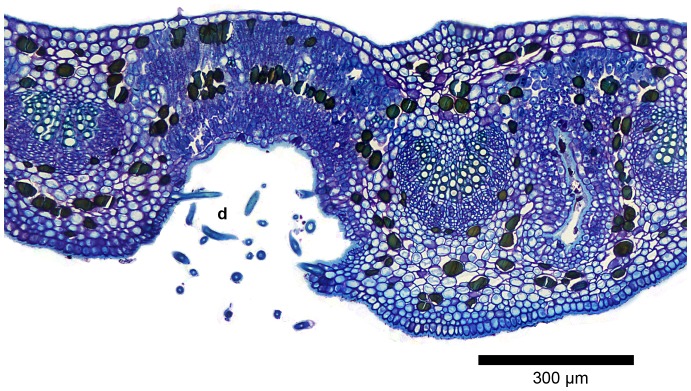
Domatium in leaf tissue. LM transverse section of a domatium and associated leaf tissue; GMA-embedded, stained with periodic acid Schiff/toluidine blue. The domatial cavity (d) extends deep within the leaf blade. The domatium is flanked by the vascular bundles of the midrib (right) and a principal lateral vein (left). A partially sectioned cavity of another domatium is present to the right of the midrib. Epidermal and mesophyll cells adjacent to the domatial cavity are not visibly modified. Darkly stained tanniniferous cells are scattered throughout the mesophyll.

**Figure 4:**
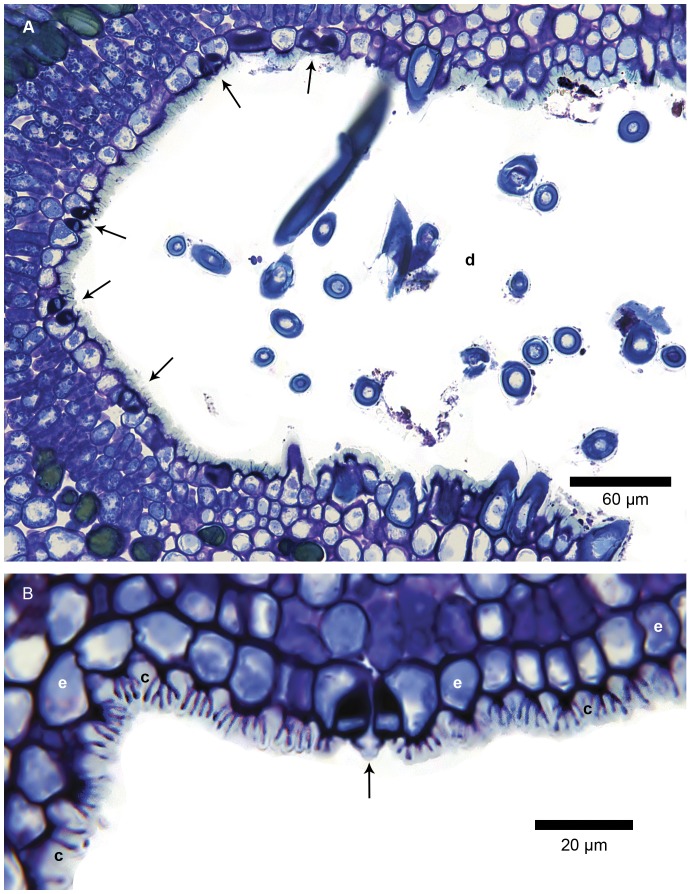
Epidermal lining of domatium. LM transverse sections of a domatium to show the epidermal lining with cuticle; GMA-embedded, stained with periodic acid Schiff/toluidine blue. (A) Hairs and stomata (arrowed) are frequent in the domatial epidermis. Hairs are concentrated towards the rim of the domatial cavity, whereas stomata are most frequent towards the inner portion. Epidermal cells have cutinised outer tangential cell walls (darkly stained) and are covered by an apparently relatively (compared to abaxial blade surface) thick cuticle traversed by faint, darkly stained, transverse lines. (B) Enlarged portion of the epidermis (e) lining the deeper parts of a domatium showing the cuticle (c) traversed by darkly stained, channel-like lines, as well as a stoma (arrowed). In 3–5 µm thick sections the true structure of these transverse lines is difficult to interpret.

The LM study revealed that the domatia form pronounced cavities, the epidermis of which bears numerous hairs especially towards the rim (Figures 3, 4A). The cavities are approximately 0.4 mm wide at their entrance and up to 0.5 mm deep, hence any one of these structures can accommodate several mites. In sucker shoots and active new growth, hairs were also present on the rest of the blade, but hairiness tends to diminish with age and in some shoots the blade is essentially glabrous from the beginning. Stomata were almost entirely confined to the abaxial surface of the leaves (hypostomatous). Large numbers were also observed in the domatia (Figure 4A).

**Figure 5:**
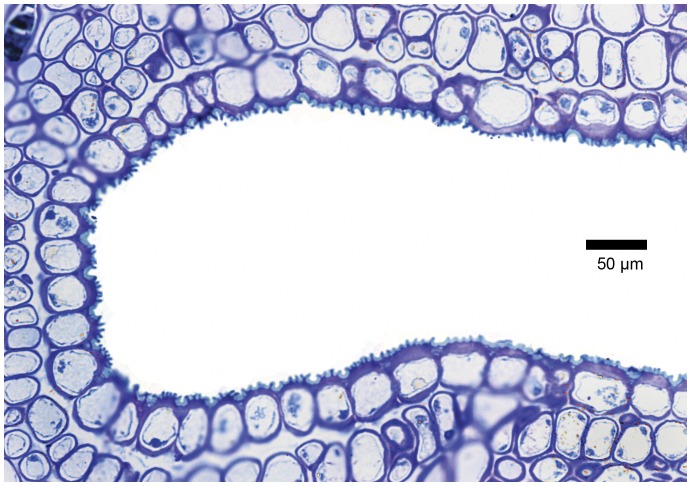
Cuticular lining of domatium. LM monitor section showing the cuticular lining in a portion of a domatium; LR White-embedded, stained with toluidine blue. In this 0.5 µm thick section it is seen that the apparently thicker cuticle (compared to non-domatial abaxial leaf surface) is due to regular, often closely-spaced cuticular folds. Furthermore, what appear as channel-like structures in the cuticle when thicker sections are view under LM (compare Figure 4), are in reality the central portion of these cuticular folds.

Under LM and utilising the relatively thick GMA sections the cuticle of the epidermis lining the domatia seemed thicker than elsewhere on the leaf. Moreover, structures resembling channels were observed extending from the outer periclinal cell walls of the epidermal cells towards the outside environment (Figures 4B, 5). These structures were far more numerous and conspicuous in the domatia than on the abaxial and adaxial leaf surfaces.

**Figure 6:**
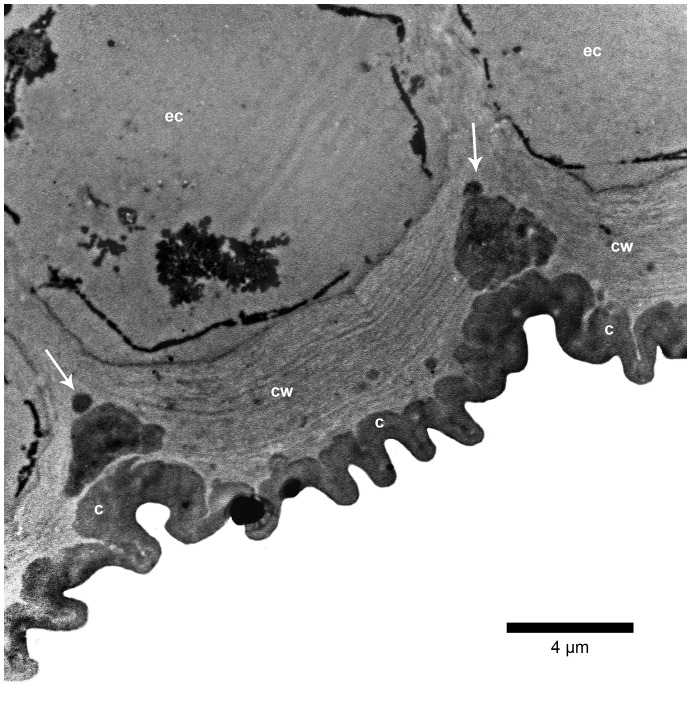
Ultrastructure of domatial epidermis with cuticle. TEM image of a portion of the epidermal cells (ec) with cuticle (c) of a domatium, showing the pronounced and regular folding of the outer surface. Large amounts of electron dense cuticular material (arrowed), which presumably would contribute to the expansion of the cuticle, are visible where adjacent epidermal cells meet (cw, outer tangential epidermal cell walls).

**Figure 7:**
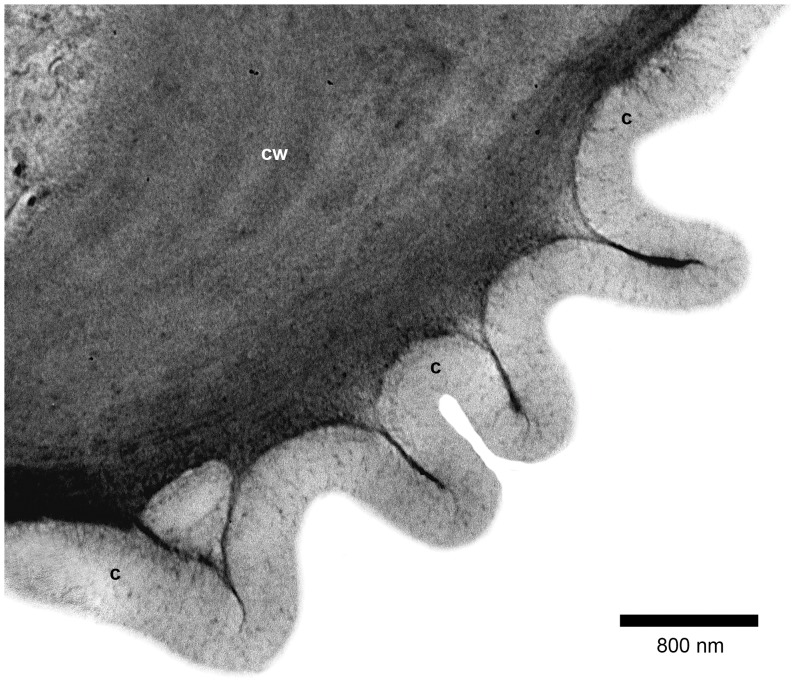
Ultrastructure of domatial cuticular folds. TEM image of a portion of an epidermal cell and cuticle (c) of a domatium, showing cuticular folds, each with a central core of electron dense fibrils extending from the underlying outer tangential epidermal cell wall (cw).

**Figure 8:**
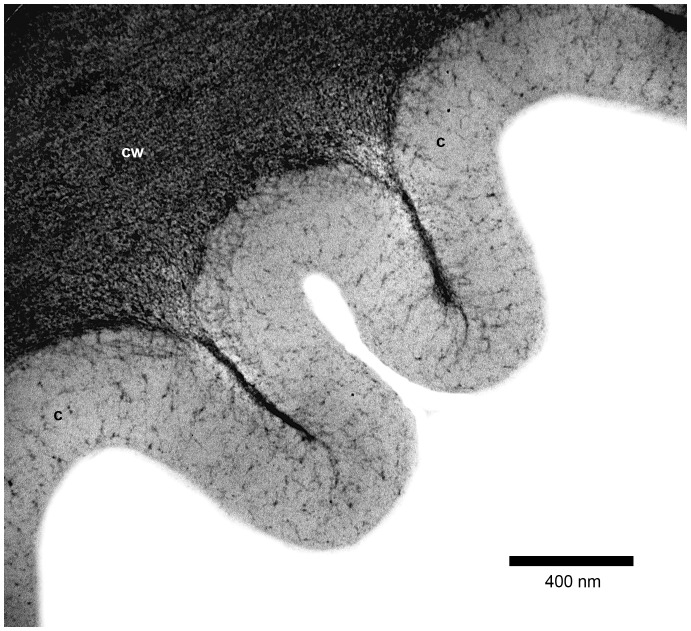
Fibrillar pattern in domatial cuticle. TEM image of a portion of an outer tangential epidermal cell wall (cw) and cuticle (c) of a domatium to show electron dense fibrillar structures branching extensively from the central cell wall extensions of the cuticular folds towards the outer surface of the cuticle.

**Figure 9:**
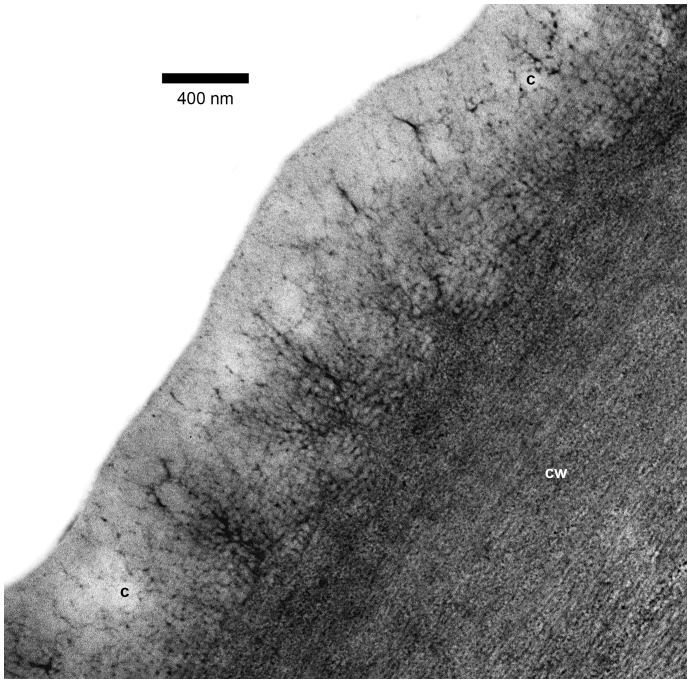
Structure of non-domatial leaf cuticle. TEM image of a portion of an outer tangential epidermal cell wall (cw) and cuticle (c) of the abaxial leaf epidermis, for comparison with those of the domatial surface. The cuticle is not folded and is traversed by a more diffusely branched system of electron dense fibrillar material.

By means of TEM (supplemented by LM using the much thinner monitor sections), the cuticle of domatia was seen to be highly folded (Figures 5, 6), thus accounting for its apparent greater thickness under LM. In addition, the structures which had the appearance of channels under LM were found to be electron dense fibrillar material contained within pronounced, essentially regular, often closely-spaced cuticular folds (Figures 6, 7). These structures branch extensively into probably single fibrils toward the outer surface of the cuticle (Figure 8). Where two adjacent cells meet, and to a lesser extent elsewhere, large amounts of cuticular material which would be used to expand the cuticle may be visible (Figure 6). This indicates that the folds of the cuticle are most probably formed by the addition of material rather than by compression. Further evidence for this is provided by the normal appearance of the cell wall underneath the folds (Figure 6). Outside domatia the cuticle is essentially smooth, with only the occasional fold. In these parts it lacks the dense fibrillar structures/extensions seen on the surface of the domatial cavity, but instead is only pervaded by a diffuse network of electron dense fibrillar material (Figure 9).

## Discussion

Mites of the family Tydeidae (broadly defined), as observed in *P. armata*, are commonly associated with domatia [4]. The group has evolved a diversity of feeding habits. Most are fungivorous, but others are predators, phytophagous or scavengers [9]. No obvious damage to the leaves of *P. armata* caused by mites was noted. It is assumed that the mites associated with the domatia of this species are most probably fungivorous or predatory and therefore beneficial to their host.

Domatia may provide a microclimate enabling mite-plant associations to be formed that would otherwise not be possible [4], [6]. This is because the survival, growth and reproduction of many species of mites are affected by changes in relative humidity that occur on the exposed leaf blade. This may well be the position in *P. armata*, especially as large numbers of stomata were found in the domatia through which water vapour could be released. Hairs are mainly associated with the domatia and would assist in maintaining a moist environment within the cavity. The possibility also of the stomata facilitating the uptake and release of carbon dioxide and oxygen may also be applicable in *P. armata*.

As early as 1896, Hamilton [10] carried out a detailed anatomical study of domatia which included seven genera and 21 species of the Rubiaceae. He found the cuticle of the domatial epidermal cells to be similar to that elsewhere on the leaf or, occasionally, thinner or thicker, but otherwise unremarkable. Similar findings have been reported in later LM anatomical studies such as those of O’Dowd and Willson [6]. We could find no reports of an apparently unusually thick cuticle in domatia such as we observed with LM.

Moraes et al. [11] studied the leaf blade anatomy and ultrastructure of six species of *Simira* (Rubiaceae). They found in all these species that the outer periclinal epidermal cell walls were composed of three distinct layers: “the inner polysaccharide-rich layer mainly composed of cellulose, the intermediate cuticular layer, with a tree-like polysaccharide-rich network immersed in a matrix of cutine (sic), and the cuticle proper”. Our TEM findings of branching fibrils appear to be similar to the “tree-like network” described by them and other workers e.g., [12] and are therefore assumed to be composed of polysaccharides. No comments were, however, made by Moraes et al. [11] relating to a possible function. In the case of nectaries (in plant families other than Rubiaceae), rather similar fibrillar strands in the cuticle have been interpreted as microchannels through which nectar is presumably secreted ([13], [14]). We would ask whether these fibrillar networks within the cuticular membrane of the domatia could not be pathways for transport.

Folding of cell walls and membranes at ultrastructural level is usually functionally associated with an increased surface area to facilitate active exchange of compounds/metabolites. The early hypothesis put forward by Lundström [5] that mites in domatia may provide plants with nutrients seems to have received little attention and support. O’Dowd and Willson [6], in their studies, found the epidermis of domatia to be unspecialised. This corroborated the findings of Stace [7]. They pointed out that secretory and absorptive processes in plants are commonly mediated by glandular structures of epidermal origin although they did not discount the possibility that they can simply occur across the epidermis. However, besides a lack of cytological or structural evidence, they failed to detect any special enzyme activity in tissues associated with domatia that would be expected if animal products were broken down and absorbed. Nishida et al. [15] similarly found no specialised structures in their study on the anatomy and development of different shapes of domatia in *Cinnamomum camphora* (Lauraceae) to support Lundström’s hypothesis. They also made no mention of any special features of the cuticle. Since it is known that cuticles are semi-permeable [16]–[18], it seems possible that exchanges, facilitated by the branching fibrils associated with the folds, would be possible.

Radioisotope feeding experiments such as on the ant-plant *Myrmecodia* cf. *tuberosa* (Rubiaceae) [19], have indicated that minerals and possibly organic substances from the ant waste may indeed be transferred into the plant. In the light of studies such as this, O’Dowd and Willson [6] suggested that similar experiments be done although they doubted “that a nutritional function is fundamental to most mite-domatia associations”.

We would question the function of the cuticular folds in the domatia of plants such as *P. armata* if some sort of exchange were not possible. This may not necessarily relate to nutrient transfer but perhaps to some other form of exchange or communication. It is, for example, now well established that plants emit diverse blends of volatile compounds from their leaves, some of which could affect the behaviour of mites (e.g., [3], [20], [21]). Compounds released by domatia may, for example, serve as cues to attract mites to these structures.

In addition to its academic interest, better understanding of domatia–mite interactions may lead to ways of manipulating morphological and chemical attributes of crop plants for a more sustainable and balanced control of insect pests in agro-ecosystems [22]. Elsewhere in Rubiaceae, Matos et al. [23] provided evidence suggesting that in *Coffea arabica* and *C. canephora*, leaves with more domatia harbour more beneficial mites. This was, however, refuted by a subsequent study on *Coffea arabica* which seems to indicate that domatia blockage treatment had no influence either on mite abundance or leaf damage in plants of this species [24]. Such contradictory reports serve to emphasize the need for more studies on the role of domatia in plant–mite interactions.

The presence of large numbers of stomata and hairs in the domatia of *P. armata* may be important in providing a suitable environment for the tydeid mites with which they are associated. The very pronounced and somewhat regular folding of the cuticle in the domatia of this taxon, reported here for the first time, most likely has some functional significance. Folding is usually associated with an increase in surface area facilitating exchanges of substances. The cuticle is expanded by the addition of material particularly from the area between adjacent cells and not from compression. The branching non-cellulosic fibrils which extend across the cuticle towards the outer surface could provide a pathway for the exchange of materials or some other form of communication between mites and plant. This therefore suggests a far more active role for the leaf in the symbiotic relationship than was previously thought. Further study is required to test such a possibility.

## Supporting Information

Video S1 **Domatium showing an associated mite.** A single tydeid mite moving in the vicinity of a domatium. Note numerous whitish mite exoskeletons in front of the domatial opening. These were shed during moulting of immature stages and are indicative of a long occupancy of this domatium by these organisms.
(MP4)
